# Notes of an European Tour

**Published:** 1845-08-01

**Authors:** F. H. Hamilton

**Affiliations:** Professor of Surgery in Geneva Medical College


### BUFFALO MEDICAL JOURNAL

**Vol. 1.** AUGUST 1, 1845. **No. 3.**

### Notes of an European Tour.---No. 3.

Communicated for the Buffalo Medical Journal, by F. H. HAMILTON, M. D., Professor of Surgery in Geneva Medical College.

DEAR M.—

To-day the hour after dinner has passed as usual, and my few American friends who generally lounge with me, have smoked and talked, and laughed, and dispersed; some to Mabille's, some to the Opera, some to the delicious Gardens of the Luxembourg close by, others to the "Elysian Fields" and Gardens of the Tuillerries across the Seine. Mindful of my promise, I have resisted my inclination to ramble also; and have remained in my "apartment meuble" to write: but barren as my tour has thus far been of "incidents," and promises still to be unless some unexpected providence interpose, I feel alarmed lest I should write more than you will patiently read. And of this particular letter I advise you beforehand that it will probably not contain one single "thrilling adventure," or "moving accident by flood or field," and if you are not in mood for plain didactics and dry statistics, I will thank you to lay it aside for a rainy day.

Of the organization of the Medical Schools in France, I have first somewhat to say. The whole system of Literary and Scientific instruction, if not perfect, is at least in my opinion unrivalled, as doubtless it ought to be, since it was established in almost precisely its present form by the great Napoleon.

Beginning at the apex of the Literary Pyramid—the "Minister of the Interior," presides over the University of France: the University is composed of a council and grand Master, or Minister of Public Instruction, in all ten persons. This body constitutes the highest appeal, and without its consent, no college, academy or primary school can be established. It employs twenty-two "Inspectors General" of studies. It has a library at the Sorbonne, that venerable and sombre building in the "quartier latin," where also free lectures are daily given. Subordinate to the university are twenty-eight academies located in the principal cities of the kingdom. Each academy is composed of several faculties or schools — in twenty-two of which are medical faculties regularly organized, and by whom lectures are given—nineteen are called "secondary" since they cannot confer the degree of Doctor in medicine—this power being reserved to the schools at Montpelier, Strasbourg and Paris.

The academy of Paris in addition to its faculty of Medicine, has also faculties of Law, Theology, Science and Letters.

The Faculty of Medicine occupies the "Ecole de Medicine," and is composed of eighteen chairs and twenty-six professors, two professors being occasionally allotted to the same department.

The base of the "Pyramid" extends much deeper, and may be traced through the several other faculties down to all the elementary schools, upon which the whole of the vast superstructure rests.

The duties of the Medical Professors consist in giving lectures at the amphitheatre of the Ecole de Medicine, or cliniques at either Hotel Dieu, La Charite, La Pitie or l'Hopital de Clinique, according as their places and duties are specifically assigned The winter course at the amphitheatre commences the 2d of Nov. and continues to the 2d of April. Seven Professors lecture alternately, three and four on the alternate days, each giving three lectures per week, or about sixty during the five months; in all about four hundred and twenty lectures. The summer course commences the 2d of April and continues to 2d of Sept., ten Professors lecture alternately, four and six on the alternate days, giving each three lectures per week, in all six hundred lectures, making the whole amount of lectures during the year, ten hundred and twenty, exclusive of the cliniques already mentioned which are given every morning at the several Hospitals. The cliniques are four Medical, four Surgical and one Obstetric. The pay of the Professors, is from the government, and varies from 2000 to 10,000 francs. To receive the degree of Doctor in medicine, a student must, if a Frenchman, be a "Bachelor of Letters," and if a foreigner, he must satisfy the examiners by the proper diploma, that he has made such literary and scientific acquirements as are implied in France by the degree of "Bachelor of Letters." He must also present a register of his birth, and a certificate of character. Four years is the time required by law for admission to a final examination ; the two last of which must have been passed under the instruction of one of the three colleges authorized to confer degrees, but the two first may have been spent at either of the secondary medical schools. The candidate to be admitted to his final examination must present testimonials that he has undergone an examination at the commencement of every year; has registered his name and paid his inscription every three months. The whole amount to be paid by the student is as follows—

| | |
|---|---|
| 15 first inscriptions at 50 francs each. - - - - | 750 fr. |
| 16th inscription - - - - - - - - - - - - - | 35 fr. |
| 5 examinations, at 30 fr. each - - - - - - | 150 fr. |
| Examining thesis - - - - - - - - - - - | 65 fr. |
| Signing diploma - - - - - - - - - - - - - | 100 fr. |
| | 1,100 fr. |

The annual number of "eleves" is about two thousand ; although those who do not register and therefore do not pay is much greater, four or five thousand. The examinations are public and severe.

There are authorized in France also another class of Physicians, called "Officers of Health:"—To be admitted to an examination for which, six years study are required with a Doctor of medicine, or five years in any Hospital, or three years in any of the medical schools. The candidates are examined by a "Jury," formed in each "department," composed of two Doctors residing in the "department," and one Professor of a medical college. The examination is upon articles of the Materia Medica; and includes a little of Anatomy and Surgery—such as pointing out the bones mentioned upon a skeleton and indicating the uses of the several instruments in a pocket case. Expense of examination 200 francs. "Officers of Health" cannot make any capital operations without the superintendence of a Doctor, and are expected generally to serve simply as attending Physicians in the absence of the Surgeon.

"Sages Femmes" receive also a regular education under instructors and in appropriate Hospitals, and before being admitted to practice, are subjected to a rigid examination; they cannot use instruments, however, without the presence and counsel of a Doctor.

There are now registered in Paris, (and none can practise who are not registered) 1,423 Doctors, 169 Officers of Health of whom 63 are Dentists, 420 Sages Femmes; in all 2,012, or about 1 to 500 of the population.

The penalty against a layman for practicing as a Doctor without a license, may be 1000 fr.; for any Officer of Health who practices as a Doctor, 500 fr.; for a female who practices as a "Sages femmes," without a license, 100 fr. For a second offence the penalty may be doubled, and the offender imprisoned for a term not exceeding six months.

For unlawful procurement of abortion, the penalty is imprisonment and hard labor.

For the revelation of professional secrets, unless by law required, the penalty is imprisonment from one to six months, and a fine of from one to five hundred francs!

A Pharmacentist must be regularly educated and licensed; he is not permitted to bring into his shop any medicines or compounds, except such as are authorized by the French Pharmacopœa; he cannot sell a compound without an order signed by a Doctor or Officer of Health. Poisonous substances must be kept in a separate case, of which the pharmaceutist alone shall carry the key, nor can they be sold to any but known citizens, under penalty of 3000 francs. The Pharmaceutist must also keep a register upon which those who buy the "poison" shall write their name, rank, residence, the nature and quantity of the drug they have received, the purpose for which they intend to use it, and the exact date of the purchase; and if the purchaser cannot write, it shall be done by the Pharmaceutist himself, and this under a penalty of 3000 francs.

In the "Rue d'Ecole de Medicine," and not far from my establishment stands the "Ecole de Medicine," a princely edifice, devoted to the use of the faculty of medicine, inaugurated the same year in which our independence was declared, and which so far partakes of the spirit of the day, that although granted by a Bourbon, its halls, museums and all its lectures are free to every one. I should not omit however, to remind you as a Yankee, that it is not unconditional freedom, since no person is admitted to its spacious and beautiful amphitheatre, carrying a cane or other stick, and his head must be uncovered. In all the public museums, galleries, &c. of Paris, canes not only, but even Ladies' shades are excluded. They are usually left at the door in charge of a porter who gives you a card faced with a printed number and affixes a duplicate of the same to the stick, on presenting this card at your return, and paying one or two sous, the article is restored. Those who value a sous will leave their canes at home.

I have been occasionally to hear Orfila, Cruveilhier and others. The students are always orderly, evincing their preferences by their attendance or absence, and never by clamorous applause, or such pointed and tangible marks of disapprobation as in 1835 disgraced the hall of materia medica, at Philadelphia. Orfila has generally a full audience, yet if I may quote the current gossip it is less the man than his learning which constitutes him a favorite. *On dit* that he has during many years, prostituted his talents and reputation as a chemist to the conviction of all persons accused of poisoning, whether really innocent or guilty, indeed that he occupies in all criminal cases of this character a "mean between accuser and executioner :" I am especially told that he is charged with having by unfair analysis obtained the conviction of the celebrated and accomplished madame Lafarge, accused of having poisoned her husband, and who is now in prison for life. An old charge is also, lately revived,—that in 1833 by secret order of the king he with others visited the Duchess de Berri, then a prisoner in the fortress at Blaye, and that for state purposes and by force he endeavored to obtain evidence of her dishonor. Moreover he is accused of having by his influence obtained the elevation of a person, by the accusers styled a "noted empyric," to the membership of the Royal Academy of Medicine,—to the rank of Chevalier and lastly to the place of surgeon to "l'Hopital des Enfants Malades." This man is the famous Guerin, the tenotomist and a greater vivisector than Magendie, since he has cut forty-two tendons in one single "seance," in the same patient! Still other charges are rumored, but of a character so grave that without additional confirmation, I dare not repeat them.

Against some of these charges, formally made by a member at a late meeting of the academy of medicine, he has written an elaborate and perhaps satisfactory defence: For myself I cannot but think that much that is said of Orfila must be put down to a malicious desire on the part of his rivals to rob him of his laurels, for I remember that always the tallest trees are most exposed to the lightning. But whether his defence be admitted or not, these things are certain, he is a great man and a great chemist, and his opinion is and will be regarded as authority by all the courts of France for years to come, whatever to the contrary, his "confreres" may say or prove.

All the professors whom I have have heard at the Ecole de Medicine, as also most of the clinical lecturers at the hospitals, sit while speaking. It surprises me that a Frenchman so full of fire and action as he usually is, can submit to such a stupid custom, since it is much more difficult in this posture to hold the attention of the listener, and to the speaker more fatiguing, yet it *may* be more dignified.

The Museum attached to the "Ecole de Medicine" contains many rare and curious specimens, and among other things a model of Bebe, the dwarf who belonged to Stanislaus, king of Poland. In 1761 he was twenty inches high and twenty-five years old, and was to have been married to Anne Souvray, of the same age, but he died that very year. "Madame Bebe," as she afterwards called herself, lived to be seventy years old, and was only thirty-three inches high at her death; she had a sister thirty-five inches high and seventy-five years old. Had the marriage been consummated we might have had a nation of Lilliputians.

In the large collection of surgical instruments is shown the case used in the autopsy of Napoleon. By-the-by, Mr. Owen showed me in the Hunterian Museum in London, two pieces of the intestine of Napoleon, presented by O'Meara, and preserved in alcohol; they were ulcerated, yet I do not remember that the physicians in their official report of the examination spoke of ulcerations out of the stomach; nor are *these* ulcerations *cancerous*; I have therefore been reflecting whether, considering the discrepancy of opinion in reference to his case, not only before but after his death, by even those who made the examination, we have not a right to doubt the accuracy of a statement which the English government were so much interested to believe, and have so positively put forth that the Ex-Emperor died of a constitutional and hereditary disease.

Opposite the "Ecole de Medicine" is the "Hopital Clinique," devoted to surgical and obstetric cases; it contains one hundred and forty beds. The surgeon is Jules Cloquet, author of several excellent treatises on anatomy, pathology, &c Paul Dubois is surgeon-accoucher — son of the celebrated Dubois who was accoucheur to the Empress Maria Louisa: and to whom Napoleon made the often quoted and much lauded remark, in reply to the inquiry of Dubois, "what shall be done if we are reduced to choose between saving the mother or child,"—"treat her," said Napoleon "as you would treat a peasant, think only of the mother," and what else could he have said? Had either the emperor or his physician any right to sacrifice the mother for the unborn child under any possible circumstances? It conveyed a base imputation upon the humanity of Napoleon to intimate that he would pass the sentence, and it spoke not well for the heart of the "good Dubois," that he could have consented to make himself the criminal actor, the executioner, in such a drama, even if commanded by his sovereign. But perhaps Dubois knew too well the soul of his master to fear for the answer, and he was willing an opportunity should be furnished him to declare his wishes before those who knew him less. I prefer this construction.

A short distance above the "Hopital Clinique" is the celebrated "Dupuytren museum," founded lately by the council of the university in honor of Dupuytren, who left 200,000 francs for the establishment, of a professorship of pathological anatomy. This hall is open to the public every Thursday, and as it is rich in models of disease and pathological specimens, it is one of the first places in Paris which the medical tourist should visit. The collection of diseased and broken bones is especially extensive and choice. I have spent many hours in a careful examination of these, assisted by a catalogue, and have some interesting, perhaps startling facts to state. (It is but justice to the accuracy of my statements to say that I have foreborne to publish any thing except what is sustained by the printed catalogue of the museum, which can always be obtained of the porter and which I have now before me, although in my own marking of the specimens at the time of the examination, the proportion of bones well united is much less than here stated. I have also thrown the whole from my notes, into a tabular form, as being more readily comprehended and more convenient for reference.)

### FRACTURE TABLE.

| Names of bones. | Total number. | No union. | Union Fibro-cellular | Union Fibro-cellular and ossific. | Union by bone, but crooked. | Union by bone, but shortened | Both Crooked and shortened | Union nearly or quite perfect. |
|---|---|---|---|---|---|---|---|---|
| Inferior Maxilla, . | 2 | | | | 1a | | | 1 |
| Clavicle, | 12 | | | | 8 | | | 4 |
| Scapula, | 3 | | | | 1 | | | 2 |
| Humerus, | 21 | 2 | | | 8 | 2 | 6 | 3 |
| Radius and Ulna, | 2 | | | | | | | 2 |
| Shaft of Ulna, | 7 | 1 | | | 2 | | | 4 |
| Olecranon, | 2 | | | | 1b | | | 1 |
| Shaft of Femur, | 49 | 2 | | | | | 37c | 10 |
| Neck of F. extra capsular, | 12 | 2 | 1 | 2 | 1 | | | 6 |
| Neck of F. intra capsular, | 24 | 16d | 5 | | | | | 3 |
| Patella, | 8 | | 7e | | | | | 1 |
| Tibia, | 13 | 1f | | | 7 | | | 5 |
| Fibula, | 11 | 1 | | | 3 | | | 7 |
| Tibia and Fibula | 12 | | | | 4 | 2 | | 6 |
| | 180 | 25 | 15 | 2 | 36 | 4 | 43 | 55 |

a. Left condyle displaced inward.
b. United by bone, but fragment much displaced.
c. One united at nearly a right angle with the proper direction; 5 are shortened two inches and a half; one, a transverse fracture, is shortened three inches and a quarter.
d. Of these, eight are recent fractures, and eight are ancient, having formed false joints.
e. No. 205, is separated 4 inches, and No. 206, six inches.
f. False joint.

You are to remember that these bones have been collected mostly from patients treated at the Parisian Hospitals, and although I have already expressed my dissent from the practice of Velpeau, and shall find equal ground of complaint against that of Jobert, yet I am compelled to acknowledge, that taken altogether, I have in no country seen more mechanical ingenuity, dexterity and care manifested in this department of Surgery, than in Paris: in short I ought to say that in every thing which pertains to Manual Surgery, French Surgeons do always excel. The wards of Jobert and Velpeau, large indeed, but small compared with the numerous other Surgical wards, could have contributed but a very small proportion of these bones; and beside, the present practice adopted by these gentlemen is recent, so recent as to render it doubtful whether they have sent to the Museum one single specimen. Let me deprive you also of another objection which may be suggested, that "we see in this not the practice of the great French Surgeons, but rather the practice of the "Internes"." On the contrary, it is a remarkable and commendable trait of these surgeons, that they rarely if ever commit a principal dressing to an "Interne," although they are often highly qualified, and in this I shall be sustained by all who are accustomed to Parisian Hospitals.

What then shall we say? are these the results of good Surgery? Some of the limbs perhaps have been permitted by the Surgeon to follow their own "bent"; circumstances often demand this: Some may have been displaced by imprudence of the patients themselves, during or subsequent to treatment; nothing is more common: Some may be the result of unskilful and blamable treatment; we are all fallible:—but I think the large mass must be regarded as testimonials of how little the very best surgical skill and appliances can accomplish under the most favorable circumstances, in the readjustment and retention of broken bones.

I believe most Surgeons are in error in reference to the power they possess in these cases, and if you also are skeptical as to the fairness of my conclusions, you shall busy yourself in measuring and "leveling" every broken limb with which you meet, and if your observation and mine concur, you will need no farther evidence of the imperfection of this branch of our art. I say this with confidence.

Adjoining the "Musee Dupuytren" is the "Ecole Pratique d'Anatomie," one of the two public dissecting rooms of Paris; I shall refer to it again.

In this street also are assembled most of the Surgical Instrument Makers, among whom Charrier and Luer have a deservedly high reputation. Charrier has just been made member of the "Legion of Honor." Not for the battles he has fought and the banners he has taken, but for his skill in the manufacture of Surgical tools! How fallen from its ancient dignity is this

> "Star of the brave! — whose beam hath shed,
> Such glory o'er the quick and dead."

since now "the mutable, rank scented many,"—Artisans, Instrument Makers, Chemists, and even Doctors, may aspire to wear this badge of honor and nobility. Scarcely a Surgeon or Physician whose name ever rises above the walls of Paris, but now bears the title of "chevalier." Azout has received it for his ingenuity in the manufacture of "papier mache," and yesterday I was told that a certain Pharmaceutist had been presented with the decorations of the order, in reward for his discovery of a new and very excellent shaving soap!

The total number of members in France is about 50,000, of whom are 263 Physicians and Surgeons, 7 officers of Health, 3 Pharmaceutists, and 1 Dentist, in the city of Paris alone. In all this you may note the policy of the "would be citizen King." But it will not do—Louis Phillippe is unpopular and must remain so despite all his efforts to the contrary.

Charrier resides at No. 6, and Luer at No. 12 Rue d' Ecole de Medicine, and a few doors further down is No. 18, an old building occupied now in front as a "tapis franc," where upon application to the ugly looking ogresse behind the desk, I was permitted to see in the second story the "very room" in which the infamous Marat was assassinated. The room is small and occupied at present by a Medical Student, who perhaps may hope to obtain a kind of surgical inspiration, by daily and constant remembrance of her, who here successfully extirpated one of the greatest and most malignant fungus excrescences with which any nation was ever afflicted.

